# Biological impact of an enhanced recovery after surgery programme in liver surgery

**DOI:** 10.1093/bjsopen/zraa015

**Published:** 2020-12-22

**Authors:** S Gonvers, J Jurt, G -R Joliat, N Halkic, E Melloul, M Hübner, N Demartines, I Labgaa

**Affiliations:** Department of Visceral Surgery, Lausanne University Hospital, University of Lausanne, Lausanne, Switzerland

## Abstract

**Background:**

The clinical and economic impacts of enhanced recovery after surgery (ERAS) programmes have been demonstrated extensively. Whether ERAS protocols also have a biological effect remains unclear. This study aimed to investigate the biological impact of an ERAS programme in patients undergoing liver surgery.

**Methods:**

A retrospective analysis of patients undergoing liver surgery (2010–2018) was undertaken. Patients operated before and after ERAS implementation in 2013 were compared. Surrogate markers of surgical stress were monitored: white blood cell count (WBC), C-reactive protein (CRP) level, albumin concentration, and haematocrit. Their perioperative fluctuations were defined as Δvalues, calculated on postoperative day (POD) 0 for Δalbumin and Δhaematocrit and POD 2 for ΔWBC and ΔCRP.

**Results:**

A total of 541 patients were included, with 223 and 318 patients in non-ERAS and ERAS groups respectively. Groups were comparable, except for higher rates of laparoscopy (24.8 *versus* 11.2 per cent; *P* < 0.001) and major resection (47.5 *versus* 38.1 per cent; *P* = 0.035) in the ERAS group. Patients in the ERAS group showed attenuated ΔWBC (2.00 *versus* 2.75 g/l; *P* = 0.013), ΔCRP (60 *versus* 101 mg/l; *P* <0.001) and Δalbumin (12 *versus* 16 g/l; *P* < 0.001) compared with those in the no-ERAS group. Subgroup analysis of open resection showed similar results. Multivariable analysis identified ERAS as the only independent factor associated with high ΔWBC (odds ratio (OR) 0.65, 95 per cent c.i. 0.43 to 0.98; *P* = 0.038), ΔCRP (OR 0.41, 0.23 to 0.73; *P* = 0.003) and Δalbumin (OR 0.40, 95 per cent c.i. 0.22 to 0.72; *P* = 0.002).

**Conclusion:**

Compared with conventional management, implementation of ERAS was associated with an attenuated stress response in patients undergoing liver surgery.

## Resumen

**Antecedentes:**

El impacto clínico y económico de los programas de recuperación intensificada después de la cirugía (*enhanced recovery after surgery*, ERAS) ha sido ampliamente demostrado. Sin embargo, no está claro si el ERAS también tiene un efecto biológico. Este estudio tuvo como objetivo investigar el impacto biológico de ERAS en pacientes sometidos a cirugía hepática.

**Métodos:**

Análisis retrospectivo de pacientes sometidos a cirugía hepática (2010-2018). Se compararon los pacientes operados antes y después de la implementación de ERAS (2013). Se monitorizaron marcadores surrogados de estrés quirúrgico: recuento de glóbulos blancos (*white blood cells count*, WBC), proteína C reactiva (*C-reactive protein*, CRP), albúmina (Alb) y hematocrito (Hct). Sus fluctuaciones perioperatorias se definieron como valores Δ, calculados en el día postoperatorio (*postoperative day*, POD) 0 para ΔAlb y ΔHct y POD 2 para ΔWBC y ΔCRP.

**Resultados:**

Se incluyeron un total de 541 pacientes, con 223 y 318 pacientes en los grupos no ERAS y ERAS, respectivamente. Los grupos fueron comparables, excepto por tasas más altas de laparoscopia (25 frente a 11%, *P* < 0,001) y de resecciones mayores (48% *versus* 38%, *P* = 0,035) en el grupo ERAS. Los pacientes del grupo ERAS mostraron una disminución de ΔWBC (2 *versus* 2,75 G/L, *P* = 0,013), ΔCRP (60 *versus* 101 mg/L, < 0,001) y ΔAlb (12 *versus* 16 g/L, *P* < 0,001). El análisis de subgrupos para la resección abierta mostró resultados similares. El análisis multivariable identificó a ERAS como el único factor independiente asociado a elevación de ΔWBC (razón de oportunidades, *odds ratio*, OR, 0,65; i.c. del 95%, 0,43-0,98; *P* = 0,038), ΔCRP (OR, 0,41; i.c. del 95%, 0,23-0,73; *P* = 0,003) y ΔAlb (cociente de riesgos instantáneos, *hazard ratio*, HR, 0,40; i.c. del 95%, 0,22-0,72; *P* = 0,002).

**Conclusión:**

En comparación con el manejo convencional, el ERAS se asoció con una respuesta atenuada al estrés en pacientes sometidos a cirugía hepática.

## Introduction

Implementation of enhanced recovery after surgery (ERAS) programmes in digestive surgery has been associated with substantial clinical and economic benefits^1–6^. Whether the application of ERAS protocols also induces a biological modulation of the stress response remains to be demonstrated, as data on the biological effect of ERAS programmes remain sparse. As ERAS implementation reduces complication rates and length of stay, it was assumed, or even speculated, that the physiological stress induced by surgery may also be reduced. Most studies have analysed cohorts of patients undergoing laparoscopic colorectal surgery, and included restraint panels of biomarkers, essentially C-reactive protein (CRP) and interleukin (IL) 6^7^.

This study aimed to determine the biological impact of an ERAS programme by analysing and comparing biomarkers of stress response in patients undergoing liver surgery before and after ERAS implementation.

## Methods

This retrospective study was conducted in the Department of Visceral Surgery at Lausanne University Hospital (CHUV) between 2010 and 2018. The study protocol was approved by the Institutional Review Board (CER-VD #2017-01169).

### Patients, groups and the enhanced recovery after surgery protocol

Consecutive patients undergoing liver surgery during the study period were included. Patients aged less than 18 years and those with no written consent were excluded. In the authors’ institution, ERAS was initially implemented for colorectal surgery (on 20 May 2011) and subsequently for liver surgery (on 5 July 2013)^8^. Patients operated on before and after implementation of the ERAS protocol in liver surgery were included in non-ERAS and ERAS groups respectively. For additional subgroup analyses, patients in the non-ERAS group operated on before and after ERAS implementation in colorectal surgery were further divided into pre-ERAS and intermediate groups respectively (*Fig. S1*)^8^. Subgroup analyses comparing open and laparoscopic resections were also conducted.

Details of the ERAS protocol have been published previously^8^^,^^9^. Briefly, it includes preoperative, intraoperative and postoperative items such as preoperative counselling, carbohydrate drinks, prevention of hypothermia, no routine abdominal drainage or gastric tube, early mobilization, and systematic audit.

### Biomarkers and endpoints

A panel of blood biomarkers including white blood cell count (WBC)^10^, CRP^10^^,^^11^, albumin^12^ and hamatocrit^13^ were selected for their capacity to reflect the amplitude of a surgical stress response. Perioperative levels of these markers were monitored via blood samples analyses. Blood draws were performed daily by nurses, before and after surgery. Preoperative and postoperative fluctuations of these markers were calculated and defined as Δvalues. Time points for determining these Δvalues relied on available data from the literature: Δalbumin and Δhaematocrit were calculated on postoperative day (POD) 0 (4–8 h after surgery)^12^^,^^13^, whereas ΔWBC and ΔCRP were determined on POD 2^10^^,^^11^. For multivariable analysis, each Δvalue was dichotomized according to its median value.

### Statistical analysis

According to their pattern of distribution, continuous variables were provided either as median (i.q.r.) or mean(s.d.) values and compared with the Mann–Whitney *U* test or Student’s *t* test. Categorical variables were provided as frequencies with valid percentages, and compared with the χ^2^ test or Fisher’s exact test. The median value was used to dichotomize each Δvalue. Univariable and multivariable analyses were performed by logistic regression, integrating multiple potential confounding factors into the model. Variables with *P* <0.050 in univariable analysis were tested further in a multivariable model. Statistical significance was defined as a two-tailed *P* value below 0.050. IBM SPSS^®^ statistics 25.0 (IBM, Armonk, NY, USA) was used to perform all analyses.

## Results

ERAS and non-ERAS groups included 223 and 318 patients respectively. Patient and surgical characteristics are detailed in *Table 1*. Groups were comparable, except for higher rates of laparoscopy (24.8 *versus* 11.2 per cent; *P* < 0.001) and major resection (47.5 *versus* 38.1 per cent; *P* = 0.035) in the ERAS group.

**Table 1:** Patient and surgical characteristics

	Non-ERAS (*n* = 223)	ERAS (*n* = 318)	*P* ^†^
**Patient demographics**			
No. of women	85 (38.1)	129 (40.6)	0.593
Age (years)^*^	64 (57–72)	63 (54–70)	0.067^‡^
ASA grade III–IV	67 (30.0)	83 (26.1)	0.330
BMI (kg/m²)^*^	24.8 (22.4–27.8)	25.3 (22.8–28.7)	0.167^‡^
Smoking	61 (27.4)	93 (29.2)	0.699
Diabetes	41 (18.4)	52 (16.4)	0.564
Cirrhosis	23 (10.3)	20 (6.3)	0.106
Cancer	179 (81)	237 (74.5)	0.118
Preoperative chemotherapy	110 (50)	132 (41.5)	0.069
**Surgery**			
Laparoscopic approach	25 (11.2)	79 (24.8)	<0.001
Major resection (≥3 segments)	85 (38.1)	151 (47.5)	0.035
Additional procedure	26 (11.7)	32 (10.1)	0.574
Duration (min)^*^	285 (213–360)	282 (192–363)	0.397^‡^
Blood loss (ml)^*^	500 (300–1000)	600 (300–1000)	0.394^‡^

Values in parentheses are percentages unless indicated otherwise;

* values are median (i.q.r.). ERAS, enhanced recovery after surgery.

† χ^2^ or Fisher’s exact test, except.

‡ Mann–Whitney *U* test.

### Impact of ERAS on perioperative profiles of stress markers

**Fig. 1:**
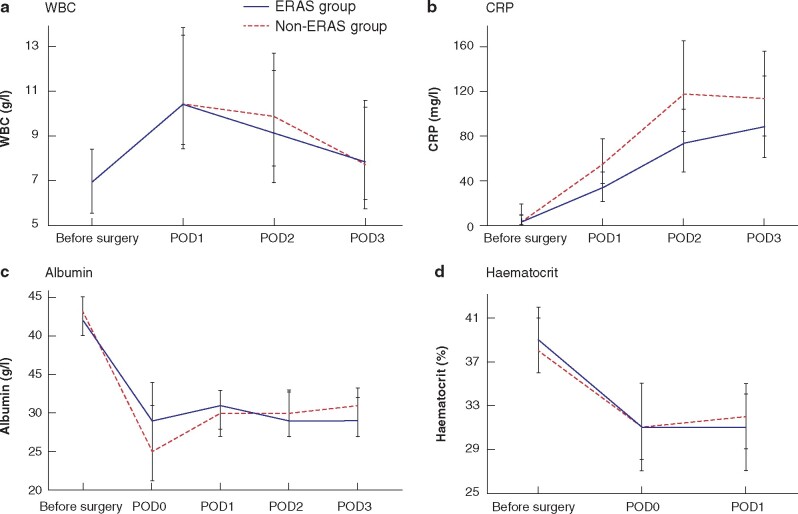
Perioperative profiles of the four biomarkers **a** White blood cell count (WBC); **b** C-reactive protein (CRP); **c** albumin; **d** haematocrit. Median (i.q.r.) values are shown.

Perioperative levels of the selected markers of the stress response were monitored. Their profiles are illustrated in *Fig. 1*.

**Fig. 2:**
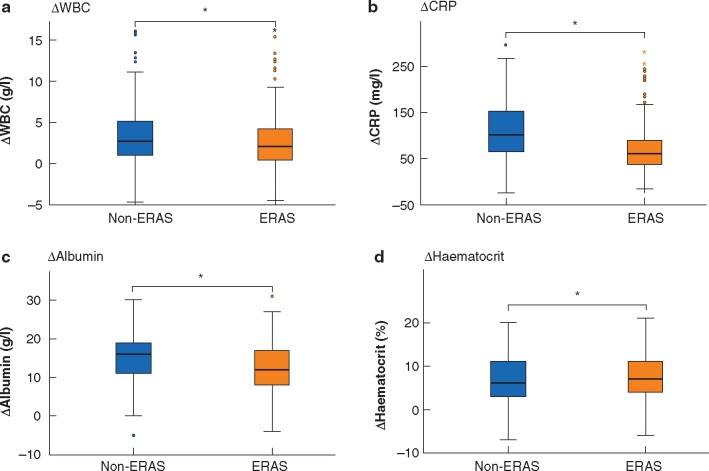
Box plots comparing perioperative markers of the stress response with and without enhanced recovery after surgery implementation Changes in **a** White blood cell count (ΔWBC) on postoperative day (POD) 2, **b** C-reactive protein (ΔCRP) on POD 2, **c** albumin (Δalbumin) on POD 0, and **d** haematocrit (Δhaematocrit) on POD 0 in enhanced recovery after surgery (ERAS) and non-ERAS groups. Median values, interquartile ranges and ranges (excluding outliers) are denoted by horizontal bars, boxes and error bars respectively. *a *P*=0.013, **b** *P* <0.001, **c** *P*<0.001, **d** *P* =0.059 (Mann–Whitney *U* test).

The difference between preoperative and postoperative values of these markers (Δvalues) in ERAS and non-ERAS groups are compared in *Fig. 2*. The ERAS group showed attenuated ΔWBC (median 2.00 g/l *versus* 2.75 g/l for the non-ERAS group; *P*  = 0.013), ΔCRP (60 *versus* 101 mg/l respectively; *P* <0.001) and Δalbumin (12 *versus* 16 g/l; *P* < 0.001), whereas Δhaematocrit showed no difference (7 *versus* 6 per cent; *P* = 0.059).

### Subgroup and multivariable analyses

Subgroup analysis of open surgery was conducted first (*Fig. S2*), and showed an impact for the ERAS protocol on ΔCRP (median 68 mg/l *versus* 108 mg/l for the non-ERAS group; *P* < 0.001), Δalbumin (13 *versus* 16 g/l respectively; *P* = 0.009) and Δhaematocrit (8 *versus* 6 per cent, p = 0.028), whereas ΔWBC was unchanged (2.4 *versus* 3.0 g/l; *P* = 0.191).

Multivariable analyses were then performed for each marker to assess the independent association between their perioperative fluctuation and potential confounders. *Table 2* summarizes the identified independent predictors of increased Δvalues for each marker. Overall, the ERAS protocol was the only factor associated with a decrease in each marker, except Δhaematocrit.

**Table 2:** Multivariable analysis of high changes in markers of surgical stress

	ΔWBC ≥2.4 g/l		ΔCRP ≥71 mg/l		ΔAlbumin ≥13 g/l		ΔHaematocrit ≥7%	
	OR	*P*	OR	*P*	OR	*P*	OR	*P*
Female sex		n.s.	0.55 (0.32, 0.94)	0.028	2.95 (1.66, 5.22)	<0.001		n.s.
Age		n.s.		n.s.		n.s.		n.s.
ASA score III–IV	1.92 (1.21, 3.05)	0.006		n.s.		n.s.		n.s.
BMI		n.s.	1.05 (0.99, 1.12)	0.101		n.s.		n.s.
Smoking		n.s.		n.s.		n.s.		n.s.
Diabetes	0.45 (0.26, 0.79)	0.005	2.43 (1.2, 4.94)	0.014		n.s.		n.s.
Cirrhosis		n.s.		n.s.		n.s.		n.s.
Cancer		n.s.		n.s.		n.s.		n.s.
Preoperative chemotherapy		n.s.		n.s.		n.s.		n.s.
Laparoscopic approach		n.s.	3.17 (1.34, 7.5)	0.009	3.24 (1.28, 8.22)	0.013		n.s.
Major resection (≥3 segments)	1.10 (0.69, 1.74)	0.689		n.s.	0.84 (0.46, 1.52)	0.562	1.07 (0.66, 1.73)	0.80
Additional procedure		n.s.		n.s.		n.s.		n.s.
Duration of surgery	1.00 (1.00, 1.01)	0.003	1.00 (1.00, 1.00)	0.670	1.00 (1.00, 1.00)	0.539	1.00 (0.99, 1.00)	0.338
Blood loss	1.00 (1.00, 1.00)	0.086		n.s.	1.00 (1.00, 1.00)	<0.001	1.00 (1.00, 1.00)	<0.001
ERAS protocol	0.65 (0.43, 0.98)	0.038	0.41 (0.23, 0.73)	0.003	0.40 (0.22, 0.72)	0.002	1.16 (0.75, 1.80)	0.502

Values in parentheses are 95 per cent confidence intervals. WBC, white blood cell count; CRP, C-reactive protein; OR, odds ratio; n.s., not statistically significant in univariable analysis; ERAS, enhanced recovery after surgery.

Finally, as the implementation of ERAS is intrinsically associated with time (patients in the ERAS group had surgery more recently than those in the non-ERAS group), it could be argued that the biological effect attributed to ERAS is due to the chronology. Subgroup analyses of the non-ERAS group aimed to exclude this bias, allowing further analysis of the pattern of markers over time. the intermediate group corresponded to patients undergoing surgery before the formal implementation of the ERAS protocol specific for liver surgery, but after the ERAS implementation in colorectal surgery (*Fig. S1*). As demonstrated previously^8^, implementation of the ERAS protocol in colorectal surgery also had an impact in liver surgery (intermediate group). A progressive biological impact was also observed in these three groups: pre-ERAS, intermediate and ERAS groups. When pre-ERAS (before any implementation of ERAS) and ERAS groups were compared, a strong difference was identified for ΔWBC (median 4.3 *versus* 2.0 g/l respectively; *P* = 0.003), ΔCRP (121 *versus* 60 mg/l; *P* < 0.001) and Δalbumin (18 *versus* 12 g/l; *P* = 0.002), whereas Δhaematocrit showed no difference (6.5 *versus* 7 per cent; *P* = 0.269) (*Fig. S3*).

## Discussion

These results suggest a biological impact for the ERAS protocol during the early postoperative phase, with an attenuated stress response in comparison to that for non-ERAS management.

Defining the design of a study aiming to explore the biological effect of ERAS is a critical and challenging step. RCTs are commonly considered to be of higher quality than retrospective studies, and should therefore be chosen whenever possible in clinical research. However, ERAS application cannot be considered as a standard treatment, so that ERAS *versus* non-ERAS and the typical ‘new drug *versus* placebo’ are not similar comparisons. This is because application of an ERAS programme is a multimodal approach that requires to be understood, assimilated and applied by a dedicated medical staff team. Teams that become familiar with ERAS management will subsequently apply its principles, consciously or sometimes unconsciously. Hence, designing an RCT comparing ERAS with non-ERAS does not preclude or reduce the risk of bias, compared with the design of a retrospective study. This point was demonstrated by a previous study^8^, which showed that ERAS implementation in colorectal surgery had a significant impact on conventional liver surgery.

The study design entailed using a comprehensive panel of biomarkers indicating the magnitude of surgical stress. These markers were selected based on their clinical relevance: they were easy to measure, commonly used in practice, reproducible, and inexpensive. In addition, Δvalues were calculated and established as the primary endpoint, as they reflect advantageously the dynamic perioperative fluctuation of these biomarkers. These Δvalues were calculated at early time points (POD 0 and POD 2) in order to show better the biological reaction during the very early postoperative phase. The cohort of 541 patients was sufficient for the required analyses, and comprised a larger sample size than in the vast majority of comparable studies^14–23^.

Thorough and comprehensive statistical analyses were performed to minimize the risk of potential bias. The two groups had different laparoscopy and major resection rates, which would have opposing consequences: the higher incidence of laparoscopy in the ERAS group may have attenuated the stress response, whereas the increased rate of major resection would rather intensify it. It may be argued that detected differences in the selected biomarkers were due to the higher rate of laparoscopic resection in the ERAS group. However, subgroup analyses of patients undergoing open resection showed similar findings. One of the strengths of the study is the multivariable analysis performed for each marker. The fact that ERAS implementation is intrinsically associated with time may be considered a potential bias, as it may be hypothesized that the delay between non-ERAS and ERAS cohorts could be associated with technical improvements and explain the biological difference detected between the two groups. The subgroup analysis of the three groups (pre-ERAS, intermediate and ERAS) provided a unique opportunity to assess the effect of the ERAS protocol over time, and showed a progressive reduction in the stress response with ERAS implementation. In addition, the similarity between the two groups (ERAS and non-ERAS) for duration of surgery, blood loss and Δhaematocrit does no support this argument.

Besides meta-analyses^7^^,^^24–26^, comparable original studies have involved smaller sample sizes. In 2012, Ren and colleagues^27^ published a large RCT including 597 patients (299 ERAS and 298 non-ERAS) in whom inflammatory biomarkers were measured: IL-1β, IL-6, interferon-γ and tumour necrosis factor-α. The study was not designed specifically to investigate the biological impact of ERAS, as the primary endpoint was length of hospital stay. In addition, although the results appeared to show a reduced stress response in the ERAS group, the selected biomarkers were of limited interest as they are not commonly used in clinical practice.

Most other comparable studies^7^^,^^14^^,^^20^^,^^23^^,^^25^^,^^26^ have analysed CRP and IL-6. Although CRP is widely monitored after surgery, IL-6 is rarely measured in daily practice, probably owing to its limited performance as well as its high cost^28–30^. Furthermore, none of these studies used Δvalues, but rather isolated values of biomarkers at heterogeneous time points.

The main limitation of the present study is its single-centre design. These results need to be validated further in an independent cohort. Of note, there is no evidence to indicate that these findings would be specific to liver surgery. Hence, they may reasonably be extrapolated to other types of surgery, although future data are needed.

From a clinical perspective, these results may help to design future studies assessing the impact ERAS programmes. Modulated biomarkers, namely ΔWBC, ΔCRP and Δalbumin, may be used as endpoints. In addition, the clinical significance of specific ERAS items may be assessed via these markers. More importantly, these results stress the need to integrate perioperative monitoring with laboratory investigations in ERAS guidelines, an important point that has been lacking to date^9^.

## Supplementary Material

zraa015_Supplementary_Data
