# Supplementary figures and images for: Biological impact of an enhanced recovery after surgery programme in liver surgery
Source: BJS Open. 2020 Dec 22;5(2):zraa015. doi: 10.1093/bjsopen/zraa015 (PMC7944514; doi:10.1093/bjsopen/zraa015)

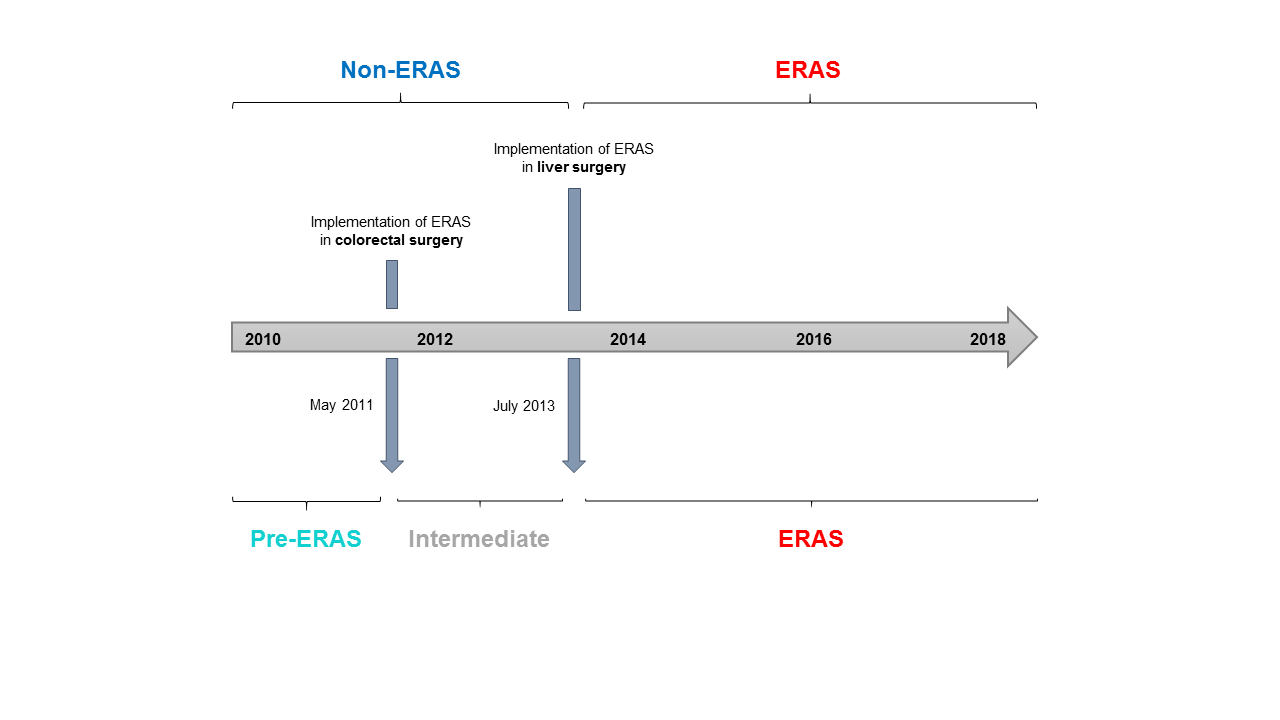

Supplement: zraa015_Supplementary_Data [file zraa015_supplementary_data.zip › suppl_data/Supplementary Figure 1.tif]

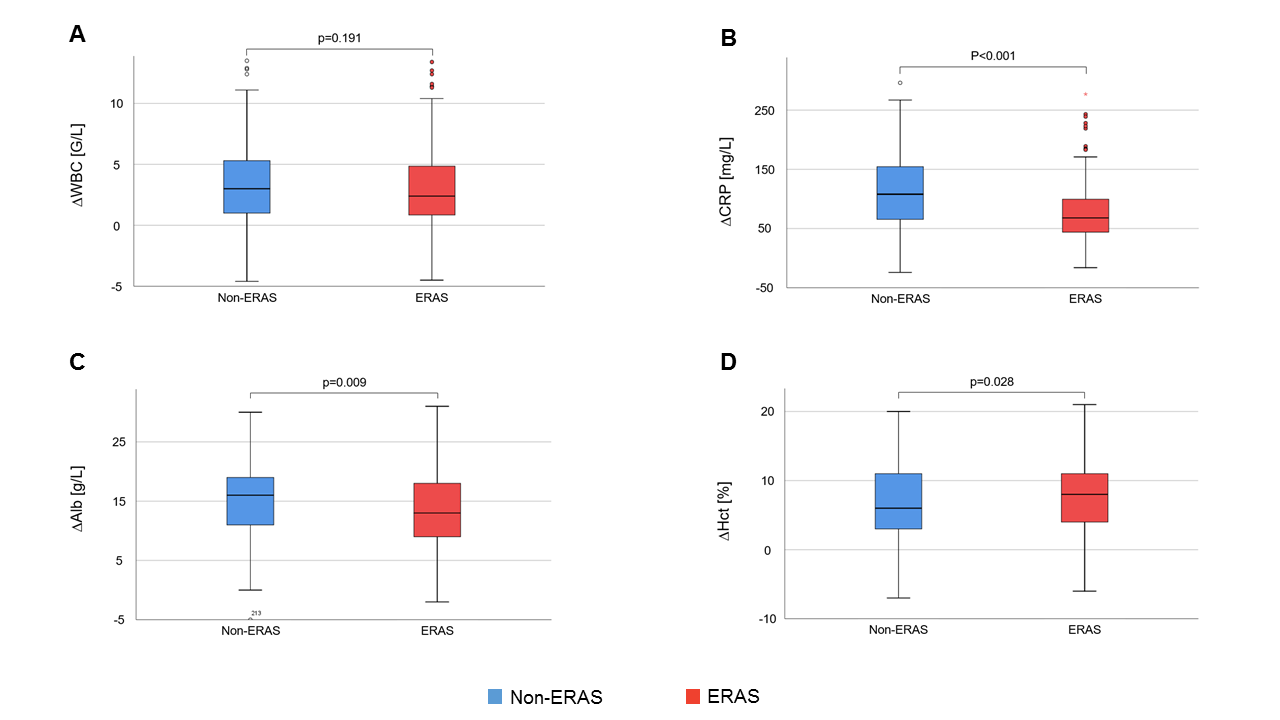

Supplement: zraa015_Supplementary_Data [file zraa015_supplementary_data.zip › suppl_data/Supplementary Figure 2.tif]

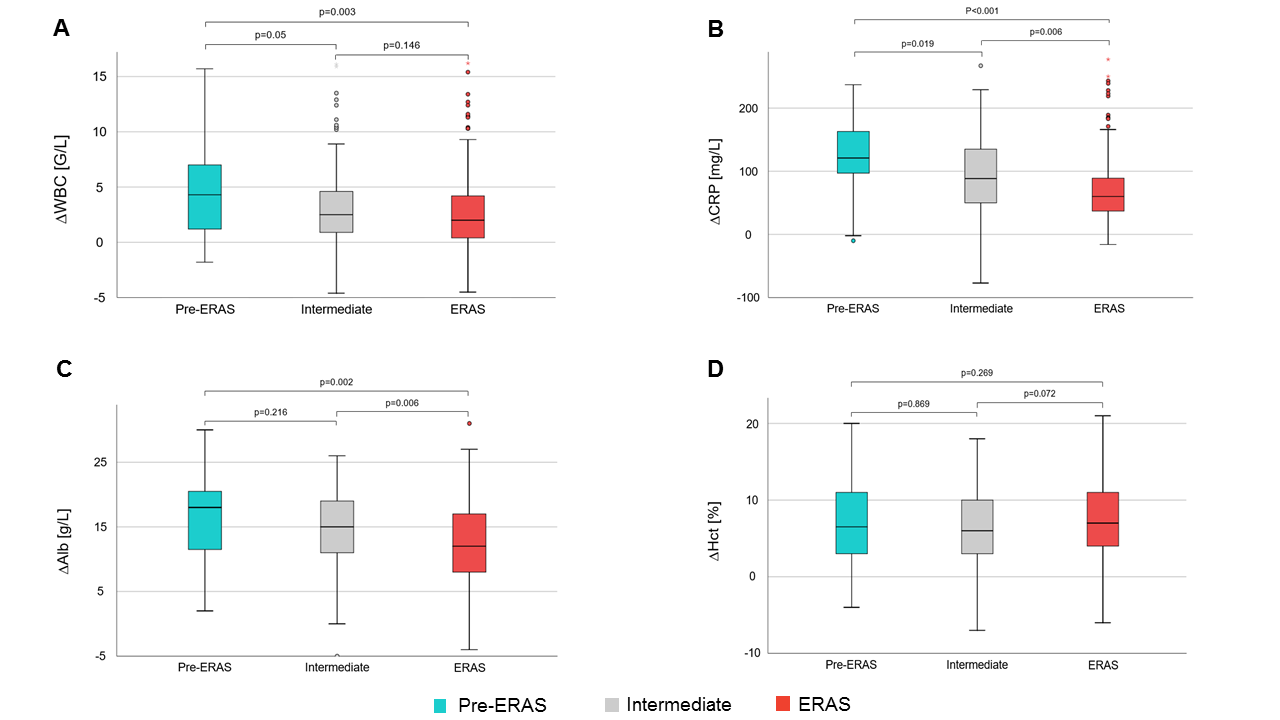

Supplement: zraa015_Supplementary_Data [file zraa015_supplementary_data.zip › suppl_data/Supplementary Figure 3.tif]
